# Transactions of the Medical Society of the State of New-York, for the Year 1829, with the Annual Address by T. Romeyn Beck, M. D. President of the Society

**Published:** 1829

**Authors:** 


Art. X. *Transactions of the Medical Society of the State of New York, for the year* 1829, *with the annual Address by* T. Romeyn Beck, M. D. *President of the Society. Albany,* 1829.

For several years the Medical Society of the state of New-York has published a report of its transactions. We have, at different times, taken pleasure in looking over these little annuals. They all, it is true, contain much that is merely local; but even this cannot be read without interest, for it proves that the physicians and surgeons of that great and populous state, are commendably labouring to organize themselves into a Profession, an example worthy of general imitation.

Among the subjects of a general nature which occupied the attention of the society, at its late session, we shall notice a few of the most prominent. These are chiefly embraced in a communication from the medical society of the city of New-York, and were submitted for the consideration of the state society.

The first of these is VACCINATION. In their communication, the committee of the district society, express themselves, in the following terms, on the causes which have led to a suspicion of the prophylactic power of vaccination.

The failures of vaccination affording protection against small pox, may be readily accounted for from the following circumstances:

1st. From the operation being performed at an improper time, as, when the child is teething or laboring under cutaneous or other diseases.

2dly. From the use of impure virus.

3dly. From peculiarity of temperament, which is not susceptible of full impression from a single insertion, but requires a second or third inoculation.

The committee then go on to recommend the establishment of a state vaccine institution, to be located in the city of New-York, and the general society concurs in the propriety of such a measure.

The city society also recommends to the state institution, to exert all its energies against the increasing and deplorable evil of INTEMPERANCE, remarking that—

It becomes the profession of medicine, whose members are so much better able to judge of the numerous instances of impaired health and premature dissolution, from the free use of ardent spirits, to proffer their efforts in discouraging so alarming and destructive an evil.

On which the state society remarked, that its attention had already been directed upon that pernicious vice.

Both societies united in the opinion, that from the increasing number of venders of medicines, called APOTHECARIES, in our cities and populous towns, and from the numerous, even fatal errors, that have arisen from the ignorance of many of them, some legislative interference is required. In this opinion we most heartily concur; being convinced that not a few of those who compound and sell medicines, are entirely unqualified for such a delicate and responsible duty. Many of them, indeed, are grossly illiterate and utterly unacquainted with the first principles of pharmaceutic chemistry.

The last subject pressed upon the state institution, by the county society, is the STEAM-DOCTORING system of one Thompson; which seems to have spread like a poisonous exhalation over the state of New-York, and even extended itself into the West, as we announced in a former number of this journal. We have not seen a more impudent, artful, and mischievous system of quackery; and, although for a long time unwilling to admit that it was in any degree worthy of the notice, or even the contempt of the profession, we have been compelled to descend from that dignified position, and acknowledge, that it exerts a sway over the minds of the multitude, that calls for the interposition of the intelligent portion of society. The annual number of its victims in New-England, New-York, Ohio, Indiana and Kentucky, is, we have no doubt, much greater than all who have died of Hydrophobia since the days of Hippocrates, and yet the man who should discover a remedy for that disease, would be ranked with the immortal Jenner.

With the laudable design of promoting discoveries and improvements in the profession, the society offers a premium of fifty dollars for the best dissertation on each of the following subjects:

1. *The history, preparation and medical uses of Iodine.*
2. *The nature, symptoms, causes and treatment of delirium tremens, illustrated by cases.*

The dissertations, we presume, to be sent in before the meeting of the society, in 1830.

To the transactoins of the society is appended the address of Dr. Beck, the president, which like every thing else from the pen of that very respectable writer, bears the impress of strong common sense, varied attainment and liberal feeling. His object, on this occasion, was to prove, that the profession is on the advance; that a sounder spirit of philosophical inquiry pervades it, than in past times; and that a more assiduous cultivation of morbid anatomy and some of the sciences collateral to medicine, has of late conducted us to many valuable improvements in pathology and therapeutics.

The following paragraph on some of the benefits which accrue to science from the efforts to overthrow and defend, the theories and opinions of eminent men, will serve to show, that the President was by no means unqualified for the task which he imposed upon himself, and with it we shall close our observations:

A subsidiary but important advantage has also accrued from the successive promulgation of these theories. The disciple, animated with an enthusiastic belief in the doctrines of his preceptor; warmed by the eloquence of his prelections, or excited by the attacks of his opponents, applies himself to the invention of new arguments, or the detection of fallacious objections. In such a contest, with much that is superfluous, and often something that is offensive, additional facts are notwithstanding elicited, and the value of old ones is better understood and more correctly applied. A rapid accumulation is thus produced, of the mass of knowledge, for which otherwise, years of desultory effort would have been necessary.—pp. 5-6.

